# Supplementary material for: Modelling Skylarks (Alauda arvensis) to Predict Impacts of Changes in Land Management and Policy: Development and Testing of an Agent-Based Model
Source: PLoS One. 2013 Jun 6;8(6):e65803. doi: 10.1371/journal.pone.0065803 (PMC3675089; doi:10.1371/journal.pone.0065803)
Supplement: Supporting Information S4 — The skylark ODdox as a zipped archive. (ZIP) [file pone.0065803.s004.zip › Skylark_ODdox/ls_8h.html]

ALMaSS Skylark ODdox: ls.h File Reference


ALMaSS Skylark ODdox
 2.0


- Main Page
- Related Pages
- Classes
- Files

- File List
- File Members

ls.h File Reference

`#include <cstdio>`  
`#include <stdlib.h>`  
`#include <math.h>`  
`#include "../BatchALMaSS/ALMaSS_Setup.h"`  
`#include "../Landscape/configurator.h"`  
`#include "../Landscape/Rodenticide.h"`  
`#include "../Landscape/maperrormsg.h"`  
`#include "../Landscape/rastermap.h"`  
`#include "../Landscape/treatment.h"`  
`#include "../Landscape/plants.h"`  
`#include "../Landscape/elements.h"`  
`#include "../Landscape/croprotation.h"`  
`#include "../Landscape/lowqueue.h"`  
`#include "../Landscape/farm.h"`  
`#include "../Landscape/calendar.h"`  
`#include "../Landscape/weather.h"`  
`#include "../Landscape/tole_declaration.h"`  
`#include "../Landscape/tov_declaration.h"`  
`#include "../Landscape/landscape.h"`  
`#include "../Landscape/pesticide.h"`


- CJT
- MSVC
- ALMaSS Working Source
- Landscape
- ls.h
- Generated on Thu Jan 10 2013 13:15:35 for ALMaSS Skylark ODdox by
   1.8.1.1
